# Genome-wide analysis of HOXC4 and HOXC6 regulated genes and binding sites in prostate cancer cells

**DOI:** 10.1371/journal.pone.0228590

**Published:** 2020-02-03

**Authors:** Zhifei Luo, Peggy J. Farnham

**Affiliations:** Department of Biochemistry and Molecular Medicine and the Norris Comprehensive Cancer Center, Keck School of Medicine, University of Southern California, Los Angeles, CA, United States of America; Università degli Studi di Milano, ITALY

## Abstract

Aberrant expression of HOXC6 and HOXC4 is commonly detected in prostate cancer. The high expression of these transcription factors is associated with aggressive prostate cancer and can predict cancer recurrence after treatment. Thus, HOXC4 and HOXC6 are clinically relevant biomarkers of aggressive prostate cancer. However, the molecular mechanisms by which these HOXC genes contribute to prostate cancer is not yet understood. To begin to address the role of HOXC4 and HOXC6 in prostate cancer, we performed RNA-seq analyses before and after siRNA-mediated knockdown of HOXC4 and/or HOXC6 and also performed ChIP-seq to identify genomic binding sites for both of these transcription factors. Our studies demonstrate that HOXC4 and HOXC6 co-localize with HOXB13, FOXA1 and AR, three transcription factors previously shown to contribute to the development of prostate cancer. We suggest that the aberrantly upregulated HOXC4 and HOXC6 proteins may compete with HOXB13 for binding sites, thus altering the prostate transcriptome. This competition model may be applicable to many different human cancers that display increased expression of a HOX transcription factor.

## Introduction

Prostate cancer is estimated to be the most common cancer type for new cancer cases and the second ranked cause of death by cancer for men in the USA [1]. A better understanding of the mechanisms that drive prostate cancer could lead to more effective cancer prevention, earlier diagnosis, and increased treatment options. Previous studies have shown an association of HOX family members with prostate cancer [2]. For example, HOXB13 controls the normal embryological development of the prostate gland [3, 4]. Studies have shown HOXB13-mediated repression of Androgen Receptor (AR) signaling, suggesting that HOXB13 may function as a growth suppressor in prostate tumors [5, 6]. In contrast, others have linked HOXB13 expression to androgen-dependent proliferation and migration in prostate cancer cells and it has been proposed that HOXB13 contributes to the development of prostate cancer by reprogramming AR binding sites [7–10]. HOXC family members are not expressed in normal prostate tissue but increased expression of HOXC genes is commonly detected in prostate cancers and multiple studies have identified HOXC4 and HOXC6 as important classifiers in panels of 3–8 genes that can be used for early diagnosis of prostate cancer, identify patients with aggressive prostate cancer, and predict recurrence of prostate cancer after treatment [11–13]. Using DNA methylation data from The Cancer Genome Atlas (TCGA), we have previously identified HOXC4 and HOXC6 in the set of top-ranked transcription factors (TFs) whose high expression correlates with the creation of prostate tumor-specific enhancers [14]. These previous findings, combined with the knowledge that decreased levels of HOXC proteins leads to decreased proliferation of prostate cancer cells [15], suggest that HOXC proteins are drivers of tumorigenesis in prostate cancer. However, there is a lack of knowledge concerning the mechanisms of HOXC-mediated gene regulation. Therefore, we have developed genome-wide binding profiles for both HOXC4 and HOXC6 and identified HOXC4- and HOXC6-regulated genes in 22Rv1 prostate cancer cells.

## Results

### Characterizing the HOXC4- and HOXC6-regulated transcriptomes in 22Rv1 prostate cancer cells

To investigate the relationship between HOXC family members and prostate cancer, we used 22Rv1 cells, which were derived from a castration-resistant human prostate carcinoma [16]. RNA-seq analysis reveals that, similar to our previous analysis of prostate tumors [2], *HOXC4* and *HOXC6* are robustly expressed in 22Rv1 cells [17]. Therefore, we have used 22Rv1 cells as our model system to study HOXC4 and HOXC6. HOXC4 and HOXC6 are very similar proteins, having 91% similarity in their C-terminal DNA binding homeodomains. Although the N-terminal regions of the two proteins are not as similar, both do have the YPWM motif thought to be critical for protein-protein interactions [18]. One important question that we wished to address is whether these two highly related HOX genes regulate the same genes. We treated 22Rv1 cells with control siRNAs or siRNAs targeting *HOXC4* and/or *HOXC6* mRNA and performed 5 independent replicates of each of 4 different treatments (5 siControl, 5 siHOXC4, 5 siHOXC6, and 5 double knockdowns); see S1 Table. With more replicates, more differentially expressed genes were identified (S1 Fig); therefore, we used all 5 replicates for each condition for our analyses.

**Fig 1:**
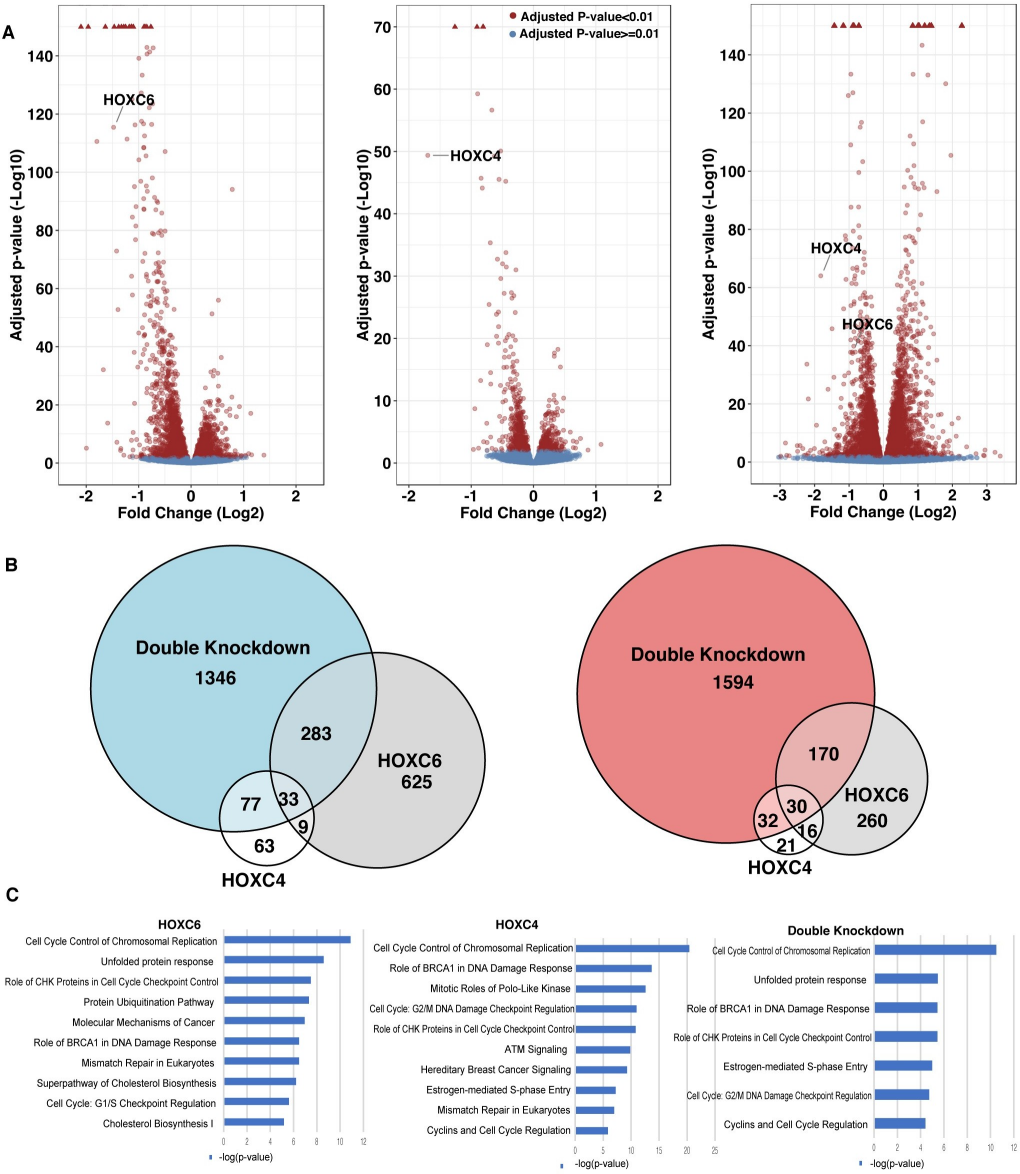
Identification of genes regulated by HOXC6 and/or HOXC4. (A) Volcano plots of genes up- or down-regulated after siRNA-mediated knockdown of *HOXC6*, *HOXC4*, or both. Red arrowheads; genes with extremely high adjusted p-values; the adjusted p-values for these genes are not shown to scale for ease of display; see S2 Table for exact adjusted p-values for all genes and S2 Fig for validation of several top-ranked genes by RT-qPCR and western blot. (B) Venn diagrams comparing the genes that are down-regulated (left panel) or up-regulated (right panel) upon knockdown of *HOXC6*, *HOXC4*, or both, using an adjusted p-value of <0.01 and a 1.2-fold cut-off. (C) Pathway analysis of down-regulated genes after knockdown of *HOXC6* and/ or *HOXC4*.

The genes differentially expressed after treatment with siRNAs to *HOXC4* and/or *HOXC6* are displayed as Volcano plots that separate the transcriptome into up-regulated vs down-regulated genes (see S2 Table for a list of all genes affected by knockdown of *HOXC4* and/or *HOXC6*). This analysis revealed that although *HOXC4* and *HOXC6* were both reduced by approximately the same amount, the number of genes affected upon *HOXC6* knockdown was greater than the number of genes affected by *HOXC4* knockdown (Fig 1A**)**. Because the DNA binding domains of the two TFs are very similar, it was possible that the genes we identified were the subset of genes that were uniquely responsive to reduction of *HOXC4* or *HOXC6*, but that we had missed a set of genes for which the two TFs play redundant regulatory functions. Therefore, we also performed a double knockdown of *HOXC4* and *HOXC6* and identified a larger set of genes that were deregulated upon simultaneous knockdown of the two HOXC TFs (Fig 1B), suggesting that these TFs have at least partially redundant functions. Pathway analysis revealed that the genes down-regulated upon siRNA knockdown of *HOXC4* and/or *HOXC6* (i.e. genes whose expression is positively regulated by the TFs in the control 22Rv1 cells) are related to cancer and cell cycle (Fig 1C); these categories were not observed for the set of up-regulated genes. These findings are consistent with a previous study showing that decreasing the levels of HOXC proteins can decrease proliferation rates of prostate cancer cell lines [15].

### Identification of HOXC4 and HOXC6 genomic binding sites in 22Rv1 prostate cancer cells

**Fig 2:**
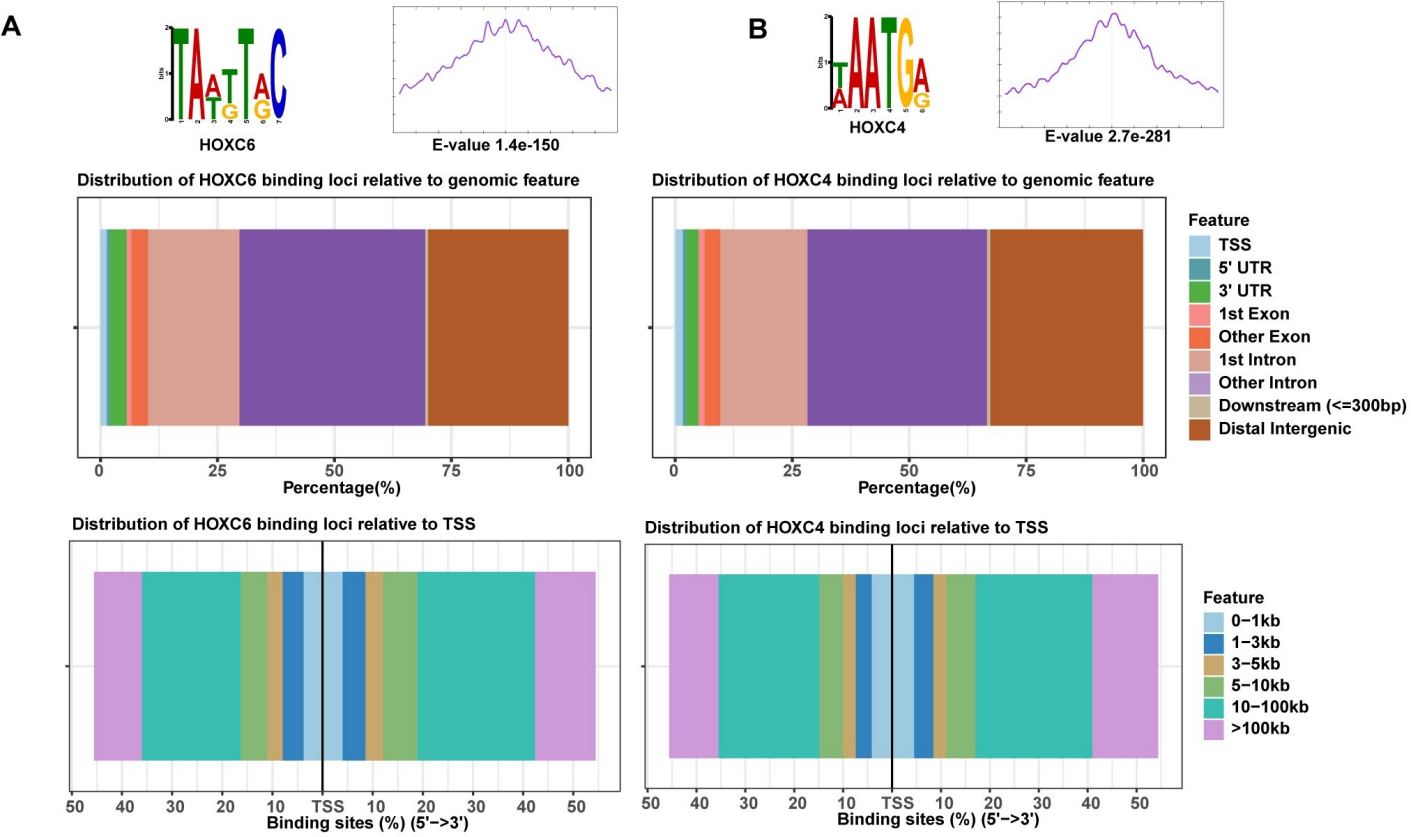
The majority of HOXC4 and HOXC6 binding sites are far from a TSS. (A) Top: Shown is the highest ranked de novo motif identified in the set of all HOXC6 binding sites; enrichment of the motif is shown within a -/+ 250bp window centered on the HOXC6 peaks. Middle: Shown is the location of HOXC6 peaks relative to gene structure. Bottom: shown is the location of HOXC6 peaks relative to distance from the nearest TSS. (B) Panels are the same as in (A) but for HOXC4 binding sites.

Because the RNA-seq analyses suggested that HOXC4 and HOXC6 may have redundant functions, we next wanted to determine their genome-wide binding profiles. We note that a genome-wide analysis of binding sites has not yet been published for either HOXC4 or HOXC6 in any human cell type and that there are very few published binding site datasets for any of the human HOX proteins. In fact, there are no ChIP-seq validated antibodies and no ChIP-seq datasets for any of the 39 human HOX TFs in the most recent ENCODE database (encodeproject.org). We obtained numerous HOXC4 and HOXC6 antibodies from a variety of commercial sources and performed ChIP-seq in 22Rv1 cells. However, no peaks were identified by ChIP-seq using any of the commercial antibodies, suggesting that it may be very difficult to develop ChIP-grade antibodies to the HOXC TFs (perhaps due to high sequence conservation). Therefore, we used Flag-tagged HOXC6 and HOXC4 proteins for our ChIP-seq experiments. We transfected 22Rv1 cells with a plasmid expressing tagged HOXC6 or tagged HOXC4, harvested the cells 96 hours after transfection, and then performed ChIP-seq using an antibody against the Flag epitope. We first performed two independent biological replicates for HOXC6; peaks were called using MACS2 and IDR was used to determine the number of reproducible peaks (see S3 Fig for our experimental and analytical ChIP-seq pipeline). We identified ~14,000 reproducible peaks for HOXC6 (see S3 Table); de novo motif analysis showed that ~73% of the HOXC6 peaks contain the canonical AT-rich HOX binding motif which is located centrally in the peaks (Fig 2A). We then annotated the HOXC6 binding sites relative to their location in a specific genomic feature. We found that the HOXC6 peaks are generally located in distal intergenic regions or in introns. We performed a similar annotation of the HOXC6 peaks with respect to the distance of the peak from the nearest transcription start site (TSS), finding that HOXC6 binds mainly to regulatory elements located farther than 10 kb upstream or downstream from a TSS. We next performed similar ChIP-seq experiments using a Flag-tagged HOXC4, identifying an AT-rich motif located centrally in the peaks and observing a similar distribution of peaks as shown for HOXC6 regarding distance from the TSS and gene structure (Fig 2B).

### HOXC6 and HOXC4 can bind to both nucleosome-free and nucleosome-occupied regions

**Fig 3:**
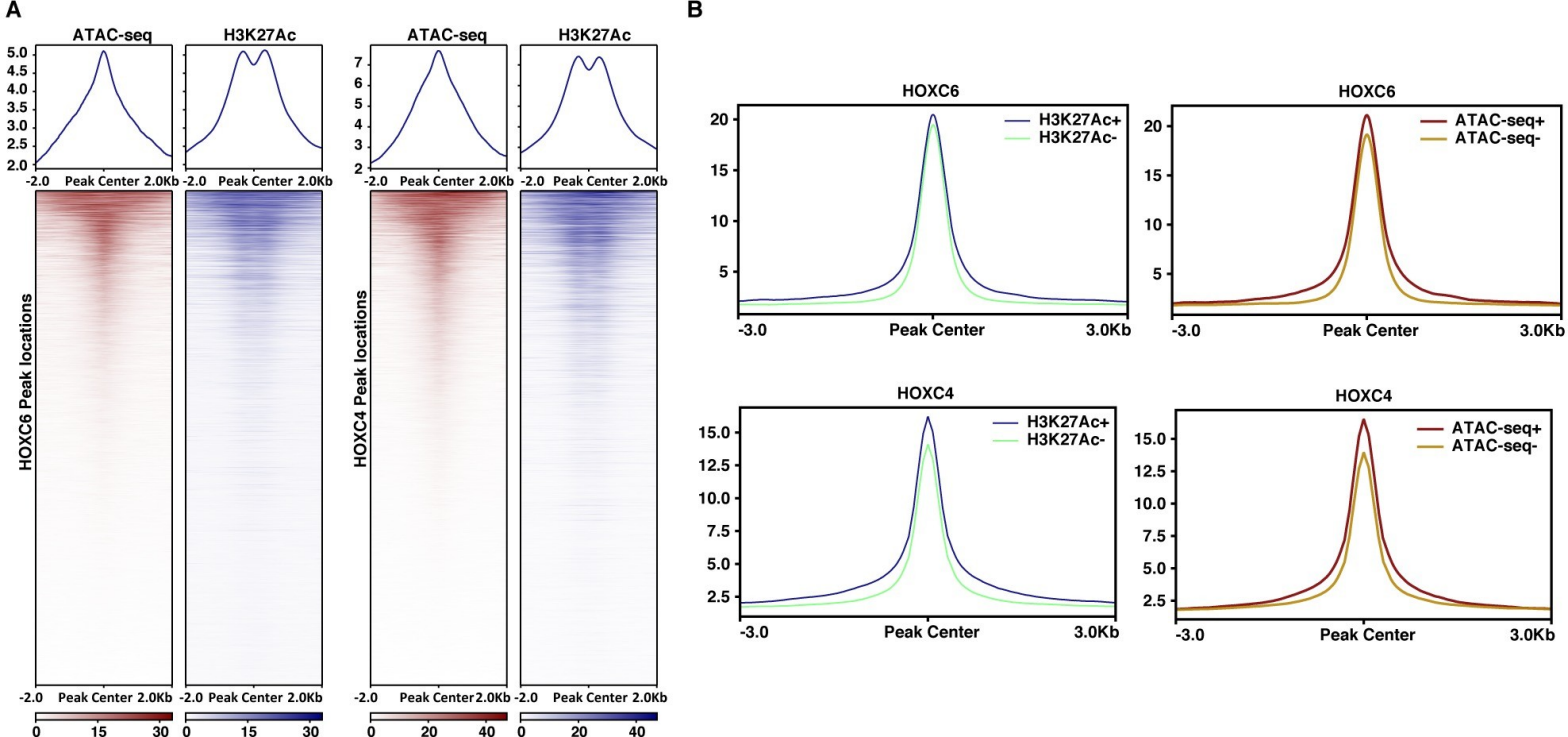
The majority of HOXC6 and HOXC4 binding sites are in nucleosome-occupied regions. (A) Shown are tag density plots and heatmaps of ATAC-seq and H3K27Ac ChIP-seq data centered on the genomic locations of the HOXC6 or HOXC4 peaks. (B) Shown are tag density plots of HOXC6 and HOXC4 peaks divided into sets that are or are not marked by H3K27Ac and sites that are or are not identified as open chromatin by ATAC-seq.

The distal binding patterns of HOXC4 and HOXC6 suggested that they may be enhancer-binding TFs. Enhancers are generally identified as regions of open chromatin that are marked by H3K27Ac. Therefore, to further characterize the HOXC binding sites, we determined if HOXC4 or HOXC6 peaks are in regions of open chromatin, using publicly available Assay for Transposase-Accessible Chromatin using sequencing (ATAC-seq) data. Surprisingly, the results revealed that the majority of the HOXC6 and HOXC4 binding sites are not in open chromatin (Fig 3A). The unexpected finding that the majority of HOXC4 and HOXC6 binding sites are not in regions identified by ATAC-seq led us to further investigate the characteristics of the peaks. Similar to the comparison to ATAC-seq, we found that the majority of the HOXC4 and HOXC6 binding sites are not marked by H3K27Ac. However, we note that the strength of binding of HOXC6 and HOXC4 is approximately equal at peaks that are H3K27Ac+ or H3K27Ac- and at peaks that are ATAC-seq+ or ATAC-seq, as noted by the similar peak heights in the tag density plots (Fig 3B), indicating that the binding of HOXC4 and HOXC6 in regions of nucleosome-occupied chromatin is as robust as the binding of the TFs at nucleosome-free, active enhancers.

### HOXC4, HOXC6, and HOXB13 have a common set of binding sites

**Fig 4:**
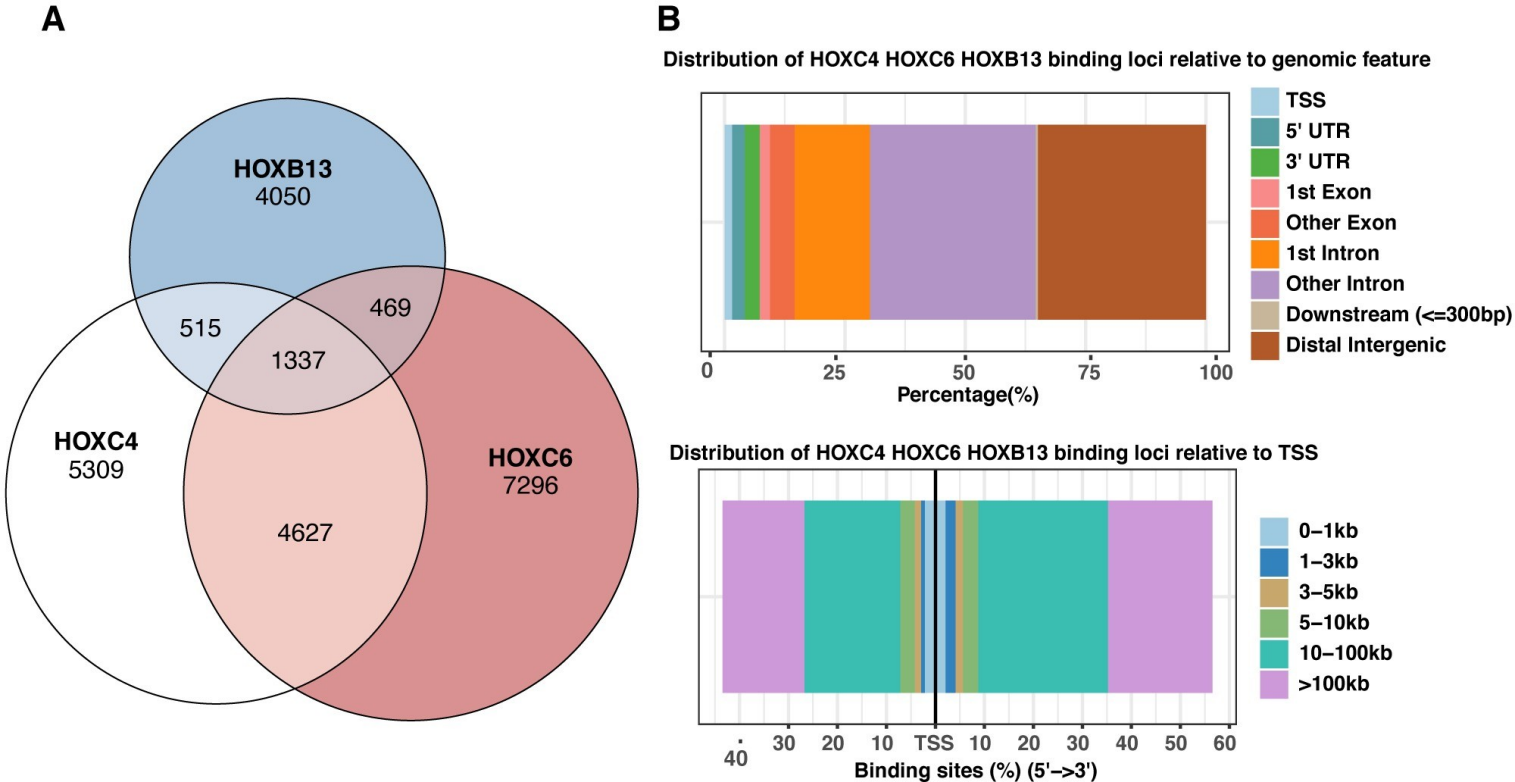
HOXC6, HOXC4 and HOXB13 bind to a common set of regulatory elements. (A). Shown is a 3-way Venn diagram comparing HOXC6, HOXC4 and HOXB13 binding sites; see also S5 Fig for additional measurements of peak overlap (B) TOP: Shown is the location of HOXC6, HOXC4 and HOXB13 co-bound sites relative to gene structure. Bottom: shown is the location of HOXC6, HOXC4 and HOXB13 co-bound sites relative to distance from the nearest TSS.

There are 39 human HOX genes located at 4 chromosomal loci [2]. These HOX genes are believed to have evolved by a series of duplication events and they have similar DNA binding domains. Previous studies have shown that different HOX genes from Drosophila can bind to similar genomic loci [18–20]. To test if this is also true for the human HOX genes that are upregulated in prostate cancer, we compared the binding sites for HOXC6 and HOXC4. The DNA binding domains of the HOXC4 and HOXC6 proteins are 81% identical and have a 91% similarity and, perhaps not surprisingly, we found that the majority of the HOXC4 binding sites overlap with the HOXC6 binding sites (**Fig 4A**). As described above, HOXB13 has also been associated with prostate cancer. Therefore, we next included HOXB13 in our comparisons (the HOXB13 DNA binding domain is 73.7% similar to the HOXC6 DNA binding domain). We first validated the specificity of a commercially available HOXB13 antibody (S4 Fig) and then performed HOXB13 ChIP-seq in 22Rv1 cells. We found that 37% of the HOXB13 binding sites are also bound by HOXC4 and HOXC6; there are a total of 2321 HOXB13 peaks shared with either HOXC4 and/or HOXC6 (Fig 4A). Characterization of the peaks co-bound by HOXC4, HOXC6 and HOXB13 revealed that the majority of the co-bound sites are within distal intergenic or intronic regions that are more than 10kb away from a TSS (Fig 4B).

**Fig 5:**
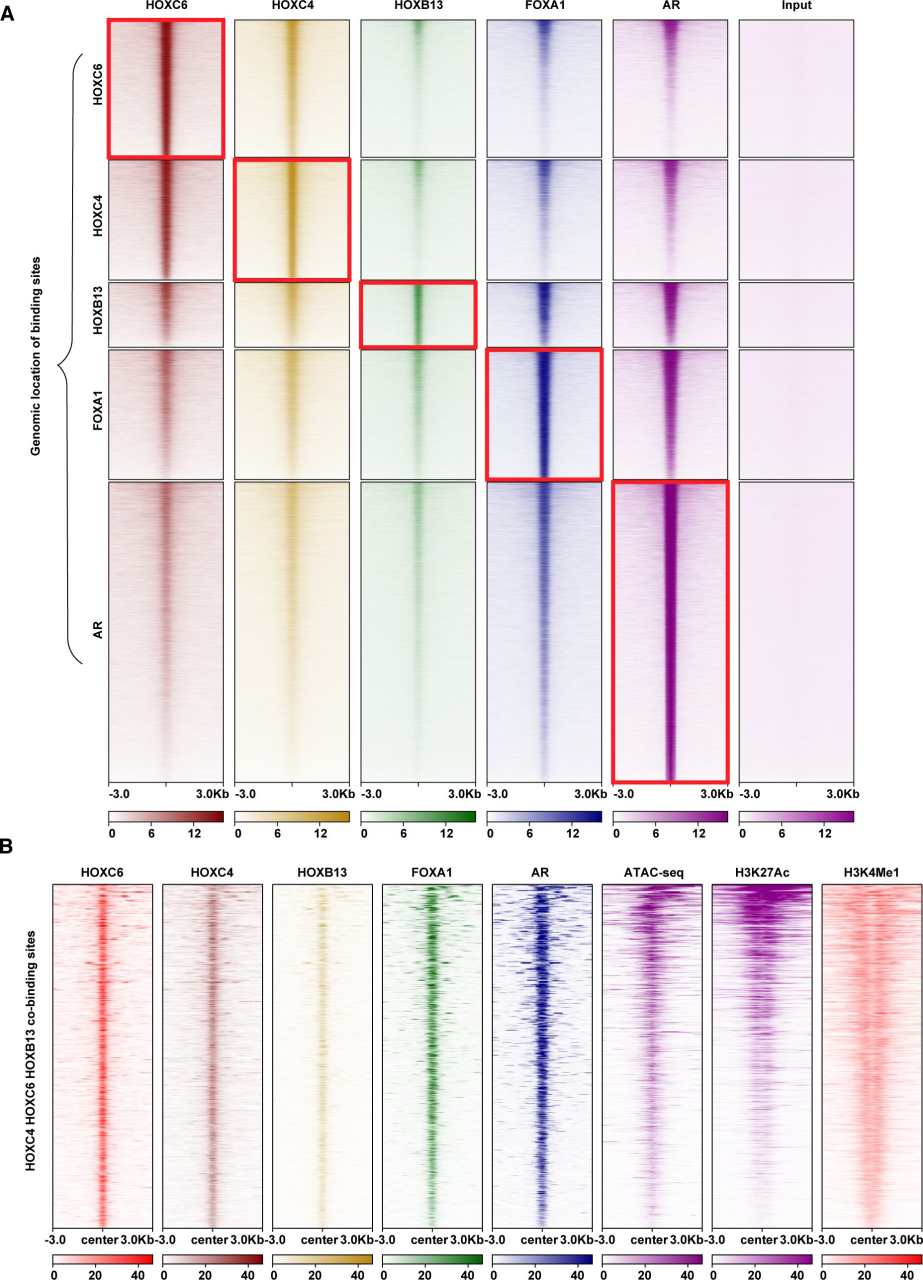
HOXC6, HOXC4, HOXB13, FOXA1, and AR co-bind to active enhancers. (A). Shown are HOXC4, HOXC6, HOXB13, FOXA1, and AR heatmaps centered on the genomic locations of HOXC4, HOXC6, HOXB13, FOXA1 or AR binding sites. Each row represents the genomic locations of the binding sites for the TF shown next to the row. The ChIP-seq tags for the 5 different factors (shown above each column) are then plotted at the genomic locations of each of the 5 factors. In each column, the red box outlines the binding sites for a given factor mapped at the binding locations for that same factor. (B) Shown are tag density plots and heatmaps of HOXC6, HOXC4, HOXB13, FOXA1, AR, ATAC-seq, H3K27Ac, and H3K4me1 data (indicated by the column heading) centered on the genomic location of the sites co-bound by HOXC6, HOXC4 and HOXB13.

Because it has been previously reported that HOXB13, FOXA1 and AR can colocalize at common binding sites, our next step was to compare HOXC4 and HOXC6 binding sites to FOXA1 and AR binding sites. We performed ChIP-seq to identify FOXA1 sites in 22Rv1 cells. To ensure high quality of our FOXA1 ChIP-seq data, we used two different FOXA1 antibodies and confirmed, using IDR, that similar sets of binding sites were detected by both antibodies. We used published ChIP-seq datasets from 22Rv1 cells for AR [21]. We found that there is a striking correlation between the strength of the HOXC4 or HOXC6 binding sites and the strength of the binding sites for the key prostate cancer transcription factors HOXB13, FOXA1, and AR (Fig 5A). However, only a subset of the binding sites is bound by all 5 TFs. Interestingly, this subset of sites is specifically the sites that are located in open chromatin marked by H3K27A (Fig 5B).

## Discussion

There is much effort directed towards the identification of markers for detection of prostate cancer. A standard screening approach is to use serum prostate specific antigen (PSA) as a biomarker for prostate cancer and, fortunately, PSA screening has reduced prostate cancer mortality rate by 20% [22–24]. However, this method falls short in accuracy and does not distinguish aggressive prostate cancer from non-life-threating prostate cancer, which can lead to overtreatment of men with localized tumors. HOXC6 and HOXC4 have been previously identified as promising, easy to assay (urinary-based) biomarkers of aggressive prostate cancer that can enhance early diagnosis and predict cancer recurrence after treatment [11–13]. The incorporation of these two genes into the screening process may lead to better clinical regimens. In addition to being good biomarkers, HOXC4 and HOXC6 may also be critical drivers of prostate neoplasia due to their ability to alter the prostate transcriptome [14]. In support of this hypothesis, studies have shown that targeted reduction in levels of HOXC6 can increase apoptosis while reducing proliferation of prostate cancer cells [15]. However, the mechanisms by which HOXC TFs influence the prostate tumor transcriptome and contribute to tumorigenesis have not been elucidated. A better understanding of the molecular function of HOXC4 and HOXC6 could provide a more comprehensive understanding of prostate cancer. Towards this goal, we identified genes that are affected by knockdown of *HOXC4* and/or *HOXC6* in 22Rv1 prostate cancer cells and performed ChIP-seq to characterize their genome-wide binding profiles.

Pathway analysis revealed that genes whose expression is positively regulated by HOXC4 and HOXC6 in prostate tumor cells are related to cancer and the cell cycle. In accordance with the hypothesis that HOX proteins are master regulators of TF pathways [25], we identified ~300 TFs that are affected in cells treated with siRNAs to *HOXC4* and *HOXC6* (S2 Table). For example, members of the E2F family, which are known regulators of the G1 to S phase transition [26–29], showed reduced expression upon knockdown of *HOXC4* and *HOXC6*. The reduction of these E2Fs supports previous studies linking HOXC6 expression to prostate cancer cell growth [15]. The oncogenic transcription regulator *YAP1* is the most significantly down-regulated gene upon the knockdown of *HOXC4*. YAP1 has been shown to promote cell proliferation and suppresses apoptosis. Thus, increased levels of HOXC4 in prostate cancer may promote tumorigenesis via upregulation of YAP1. Several of the top-ranked genes that are down-regulated upon knockdown of HOXC6, such as *EIF4G1* (which regulates a rate-limiting step in translation initiation) and *ALY/REF* (which plays a key function in mRNA export) are known to influence protein abundance. OS9, which binds to HIF-1 (a key regulator of the hypoxic response and angiogenesis) and promotes the degradation of one of the HIF-1 subunits, is also down-regulated in the *HOXC6* knockdown cells. Thus, increased levels of HOXC6 in prostate cancer may promote tumorigenesis either by enhancing overall protein levels (which would enable accelerated cell proliferation) or by supporting prostate cancer metastasis to the bone environment. The genes that show altered regulation upon knockdown of HOXC4 or HOXC6 are a combination of direct and indirect target genes. Because only a very small percentage of HOXC6 or HOXC4 binding sites are within 1 kb of a TSS, it is not possible to link the individual HOXC4 or HOXC6 binding sites to regulation of specific genes. Therefore, we cannot distinguish genes directly regulated by these HOX proteins from genes whose expression is indirectly altered (due to changes in signaling pathways regulated by the direct targets).

**Fig 6:**
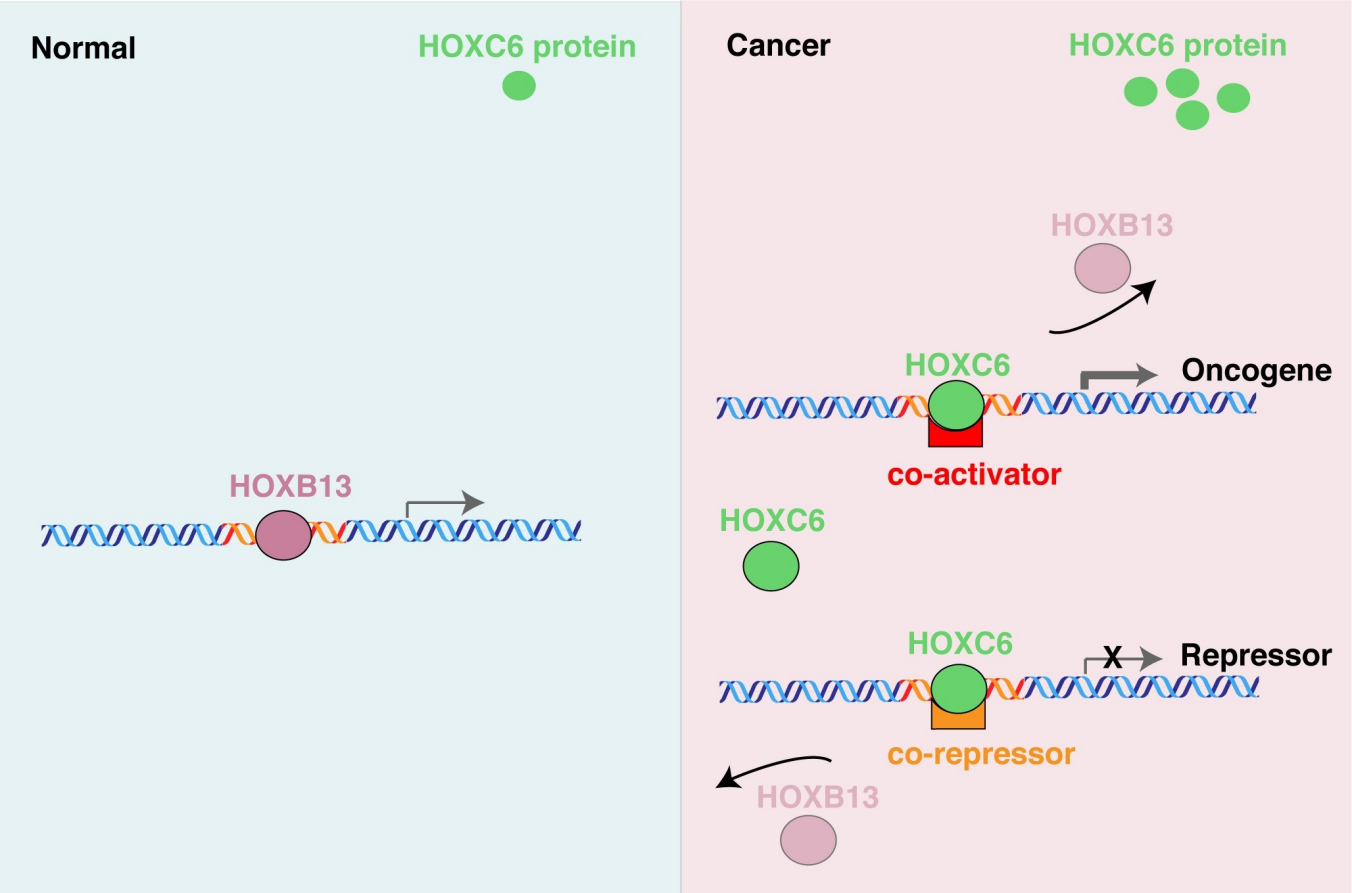
Competition model for HOX protein-mediated regulation of the prostate cancer transcriptome. Left panel: HOXB13 is expressed in normal prostate tissue, binds to its target sites, and regulates the prostate transcriptome. There are very low levels of HOXC6 in normal prostate cells and therefore this TF does not contribute to gene regulation. Right panel: HOXC6 is aberrantly expressed in prostate tumors and the high levels of HOXC6 protein compete with HOXB13 for occupancy of common binding sites. The bound HOXC6 could increase expression of an oncogene (if it recruits a co-activator) or reduce expression of a tumor suppressor (if it recruits a co-repressor).

The HOX genes are critical regulators of normal development, with different HOX genes being associated with the development of different organs. [2] Interestingly, although those studies suggest very specific functions of different HOX genes, other research has suggested that different HOX proteins can bind, with low affinity, to a similar set of binding sites. The ability to bind to the same places but produce different phenotypes is termed the HOX specificity paradox. It has been proposed that post-DNA binding actions (such as interaction of different HOX family members with distinct protein partners) are the primary determinant of normal HOX function. If so, this suggests a model for how the inappropriate expression of certain HOX genes could contribute to cancer. We hypothesize that dysregulated HOXC genes could affect tumor progression not only by creating new enhancers but also by hijacking distal regulatory elements where the normally expressed HOX genes bind in that tissue (Fig 6). In our study, we show that HOXC4 and HOXC6 colocalize with HOXB13, which is present at high levels in both normal and tumor prostate tissue. It is possible that HOXB13 (and its protein partners) regulate the normal prostate transcriptome (in the absence of HOXC4 and HOXC6, which are not expressed in the normal prostate), but when levels of HOXC4 and HOXC6 are inappropriately elevated in tumors, HOXB13 is kicked off the common binding sites causing alterations in the transcriptome. The outcome of this HOX substitution could be a combination of inappropriate activation and inappropriate repression of different genes, depending on the protein partners that are recruited by the different HOX TFs. Several co-regulators which have been shown to interact with other HOX proteins are expressed in the 22Rv1 prostate cancer cells, including PBX1-3, MEIS1 and MEIS3, and PKNOX1. However, there is no protein-protein interaction data for HOXC4 or HOXC6 in any human cell type. Future mass spectrometry studies could be useful in furthering our understanding of the mechanisms by which HOX proteins achieve specificity in the regulation of their target genes. We note that there are an increasing number of reports that associate elevated levels of HOX TFs with various cancers. In fact, every HOX gene has now been shown to have increased expression in at least one type of solid tumor [2, 30, 31]. Thus, this competition model may be relevant not only for prostate cancer but for many different cancer types.

In summary, we have shown that HOXC4 and HOXC6 regulate critical genes affecting prostate cancer proliferation. In addition, we showed colocalization of HOXC4 and HOXC6 at HOXB13 sites that are also bound by FOXA1 and AR, which have been previously linked to regulation of prostate cancer cell proliferation [9, 15, 21, 32, 33]. We suggest that the inappropriately high levels of HOXC4 and HOXC6 that occurs upon neoplastic transformation of prostate cells may contribute to tumor progression by driving oncogene expression or repressing tumor suppressor genes via binding at distal regulatory elements and altering the levels of chromatin-bound HOXB13. To accurately test this model, what is needed is a comparison of the binding of the endogenous HOXC4, HOXC6, and HOXB13 in normal and tumor prostate cells. However, there is a lack of functional antibodies for HOXC4 and HOXC6; in fact, our studies using the tagged HOXC proteins provide the first genome-wide binding profiles of HOXC4 or HOXC6 in any human cell type. Therefore, testing the proposed model will require tagging of the endogenous HOX proteins, which will be a laborious task, but will allow further insights into how HOX proteins regulate the transcriptome in normal and cancer cells.

## Materials and methods

### Cell culture

22Rv1 (CRL-2505) cells were purchased from American Type Culture Collection (ATCC) and cultured in RPMI 1640 supplemented with 10% fetal bovine serum (Gibco by Thermo Fisher, #10437036) plus 1% penicillin and 1% streptomycin at 37°C with 5% CO_2_ for less than 20 passages_._ Cell lines were authenticated via the Short Tandem Repeat method by the Norris Comprehensive Cancer Center Media Core and tested to be mycoplasma free using a universal mycoplasma detection kit (ATCC 30-1012K).

### siRNA knockdown

22Rv1 cells were transfected with DharmaFECT1 reagent (Dharmacon, #T-2001-01) plus siRNAs against human HOXC4 (ON-TARGETplus Human HOXC4 (3221) siRNA-SMARTpool, L-013462-00-0005), human HOXC6 (ON-TARGETplus Human HOXC6 (3223) siRNA-SMARTpool, L-011871-00-0005), or a non-targeting control (ON-TARGETplus Non-targeting Pool). The final concentration of siRNAs in the cell culture media was 50nmol. Cell were incubated for 24 hours and then transfected again using same amount of the same siRNAs and incubated for another 24 hours when they were harvested for analysis by RNA-seq.

### RNA-seq

RNA integrity was checked using a 2100 Bioanalyzer (Agilent technologies, G2939AA) and an RNA 6000 nano kit (Agilent technologies, 50671511). Libraries were made using the Kapa mRNA HyperPrep Kit (KAPA, KKK8581) with 500ng total RNA. Samples were sequenced on an Illumina HiSeq3000 with single-end 50 bp read length to achieve at least 30 million reads. Quality control of the sequenced datasets was performed using FastQC and reads were trimmed using Trimmomatic [34]; reads were mapped to the human genome GRCh38 reference using Gencode V29 annotation and STAR[35]. Differentially expressed genes were determined using DESeq2 after normalizing raw read counts [36]. Transcripts per million (TPM) for each gene were calculated as following with R script:

$${TPM}_{i}=\frac{x_{i}}{l_{i}}\times \left\{\frac{1}{\sum_{j}\frac{x_{j}}{l_{j}}}\right\}\times 10^{6}$$

where *x_i_* is counts for a particular gene and *l_i_* denotes its length. If a gene had less than 1 TPM in both the deletion and control datasets, then it was excluded from further analysis. The genes that showed a significant change in expression were evaluated using the Ingenuity Pathway Analysis tool (QIAGEN) for functional annotations.

### Transient transfection

15cm dishes of 22Rv1 cells were transfected using lipofectamine 3000 (Life Technologies, L3000015) with 40μg of a Flag-tagged expression vector encoding HOXC4 (GeneCopoeia, EX-A3619-M11) or HOXC6 (GeneCopoeia, EX-E1530-M14), or a corresponding control plasmid expressing eGFP (GeneCopoeia, EX-EGFP-M14); the cells were grown for 96 hours, and then harvested for ChIP-seq.

### ChIP-seq

Cells expressing the Flag-tagged HOXC4 or HOXC6 proteins (described above) were crosslinked with disuccinimidyl glutarate (DSG) (Thermo Fisher Scientific, 20593) for 45min and formaldehyde (VWR, EMD-FX0410-5) for 15min, followed by the addition of glycine to stop the crosslinking. After washing, chromatin was prepared using a cell lysis buffer (Cell Signaling, 81804), nuclear extraction, and sonication. 10μg anti-Flag antibody (Sigma, F1804-200UG) was used to immunoprecipitate 200μg of chromatin. Chromatin was pulled down using protein G beads (Thermo Fisher Scientific, 10003D), eluted from the beads using elution buffer (1% SDS, 50nM NaHCO_3_), then reverse-crosslinked overnight. ChIP-seq libraries were prepared using the Kapa Hyper prep kit (Kapa, KK8503) according to the manufacturer’s protocol. FOXA1 and HOXB13 ChIP was performed similarly as described above but without DSG crosslinking and only treated with formaldehyde for 10 minutes. 5μl anti-FOXA1 antibody (Abcam, ab5089; Cell Signaling, 58613S) or anti-HOXB13 antibody (D7N8O; Rabbit mAb, Cell Signaling, 90944) was used to pulldown chromatin. Samples were sequenced on an Illumina HiSeq3000 machine using 50bp single end reads. All ChIP-seq data were quality checked using FASTQC and MultiQC[37] and mapped to GRCh38 using BWA [38]. Low quality, duplicated, not primary aligned, and not properly paired reads were filtered out using SAMtools, Sambamb and Samblaster[39–41]. Peaks were called using MACS2[42] on each ChIP-seq replicate and common peaks were identified using IDR (for transcription factors) or the overlap method (for histones). Motifs analysis was performed using MEME tools[43]. Other ChIP-seq datasets were downloaded from the ENCODE portal (encodeproject.org) or from GEO (see S1 Table for accession numbers and S3 Fig for a schematic of the ChIP-seq pipeline).

### Western blot

Whole cell lysate was prepared using RIPA buffer (20mM Tris pH 7.4, 150mM NaCl, 1% NP40, 0.25% deoxycholic acid, 0.1% SDS) with protease inhibitors (cOmplete^™^ Protease Inhibitor Cocktail, Sigma Aldrich) and sonicated briefly using a Bioruptor Pico (Diagenode) sonicator to reduce sample viscosity. Total cellular protein was denatured at 95°C for 5min, separated on 7.5% or 12.5% SDS-PAGE gels (BioRAD, #4561025, #4561045), and then transferred to a nitrocellulose membrane. The membrane was blocked for 1 hour with 5% nonfat milk (BioRAD, 1706404XTU) in TBST (20mM Tris pH 7.4, 150mM NaCl, 0.1% Tween 20) then washed 3 times (5 min each time) with TBST. Primary antibody was added at 1:1000 dilution in TBST with 5% BSA (Sigma Aldrich, #A9647-50G) and incubated overnight at 4°C. The primary antibodies used were raised against Beta Actin (proteintech, 60008-1-Ig), OS-9 (D8P4G; Rabbit mAb, Cell Signaling, 12497), YAP (D8H1X; XP® Rabbit mAb, Cell Signaling, 14074), THOC4/ALY (D3R4R; Rabbit mAb, Cell Signaling, 12655), eIF4GI (D6A6; Rabbit mAb, Cell Signaling, 8701) and HOXB13 (D7N8O; Rabbit mAb, Cell Signaling, 90944). The next day, secondary antibodies (Thermo Scientific, Rabbit anti-Goat IgG (H+L) Cross Adsorbed Secondary Antibody, DyLight 800 conjugate, and Goat Anti-Mouse IgG (H+L), DyLight 680 Conjugated) were incubated for 1 hour at room temperature and washed as described above. Fluorophore signal was detected using the Li-COR Odyssey system and quantified using ImageJ.

### RT-qPCR

RNA was extracted using the DirectZol RNA kit (Zymo, R2062). The iScript Reverse Transcription SuperMix (Bio-RAD, 1708841BUN) was used to synthesize cDNA from 500ng RNA. The PerfecTa SYBR Green SuperMix (Quantabio, 95054-02K) was used in quantitative PCR reactions. Primers used in RT-qPCR were designed using primer-blast and listed in S4 Table.

## Supporting information

S1 Fig Increasing the number of RNA-seq replicates can identify a larger number of differentially expressed genes. (A) Shown is the number of differentially expressed genes (adjusted p-value<0.01) identified by RNA-seq using 2 to 5 replicates of control and HOXC6 siRNA-treated 22 Rv1 cells. (B) Shown is a four-way Venn diagram displaying the overlap of differentially expressed genes (adjusted p-value <0.01) identified by RNA-seq using 2 to 5 replicates of control and HOXC6 siRNA-treated cells.
(PDF)

S2 Fig Confirmation of a subset of HOXC4 and HOXC6 target genes. (A) RT-PCR analysis of HOXC4- and HOXC6-regulated genes. A second siRNA knockdown of HOXC4 or HOXC6 was performed using 22Rv1 cells. In each panel, the genes identified to be regulated by the specific HOXC protein are indicated; the genes identified as targets for the other HOXC protein are used as controls. (B) Western blot confirmation of HOXC4- and HOXC6-regulated genes; OS9, EIF4G1, and ALY/REF were identified by RNA-seq as HOXC6-regulated genes, whereas YAP1 was identified by RNA-seq as a HOXC4-regulated gene.
(PDF)

S3 Fig ChIP-seq experimental and analytical flowchart. Shown are the steps used to perform and analyze the HOXC6 ChIP-seq experiments; see Methods for details.
(PDF)

S4 Fig Validation of the specificity of the HOXB13 antibody. Shown is a Western blot demonstrating the specificity of the HOXB13 antibody; siRNA-mediated knockdown of HOXB13 mRNA eliminates the signal detected by the HOXB13 antibody.
(PDF)

S5 Fig Quantitative measures of co-binding of transcription factors. Shown are 3 tests that measure the overlap between the binding sites of HOXC6, HOXC4, HOXB13, FOXA1 and AR. The yellow number is the P-value for a two tail fisher exact test obtained using the bedtools fisher function, the red number is the Jaccard value generated using the bedtools jaccard function, the blue value is the number of overlapped peaks called using the MACS2 peak caller.
(PDF)

S1 Table Genomic datasets. (XLSX)

S2 Table HOXC6- and HOXC4-regulated genes. (XLSX)

S3 Table HOXC6- and HOXC4 ChIP-seq Peaks. (XLSX)

S4 Table Primers used in RT-qPCR and qPCR. (XLSX)
