# Supplementary figures and images for: Genome-wide analysis of HOXC4 and HOXC6 regulated genes and binding sites in prostate cancer cells
Source: PLoS One. 2020 Feb 3;15(2):e0228590. doi: 10.1371/journal.pone.0228590 (PMC6996832; doi:10.1371/journal.pone.0228590)

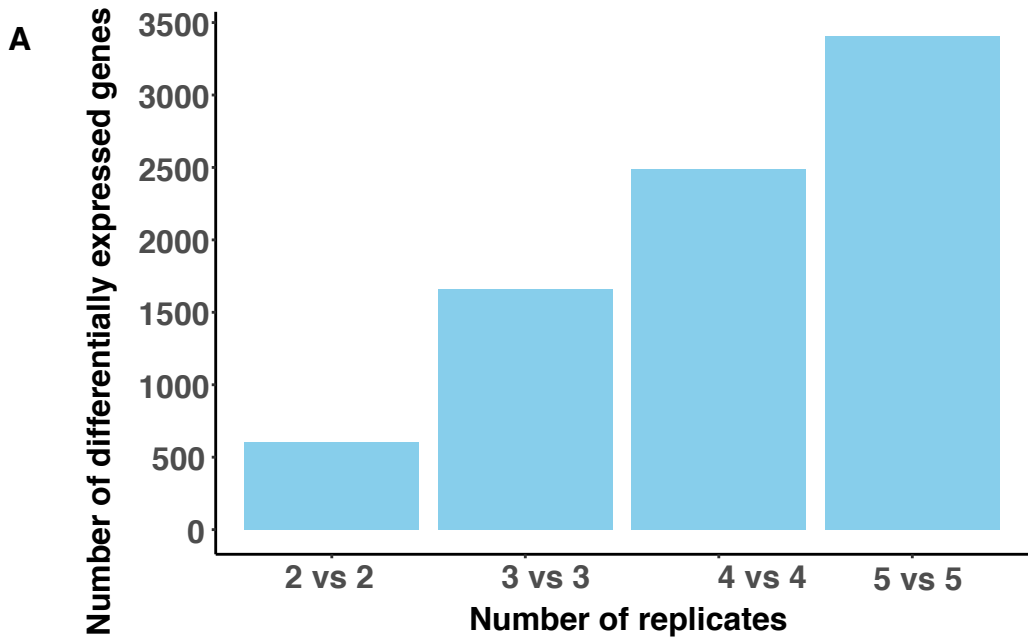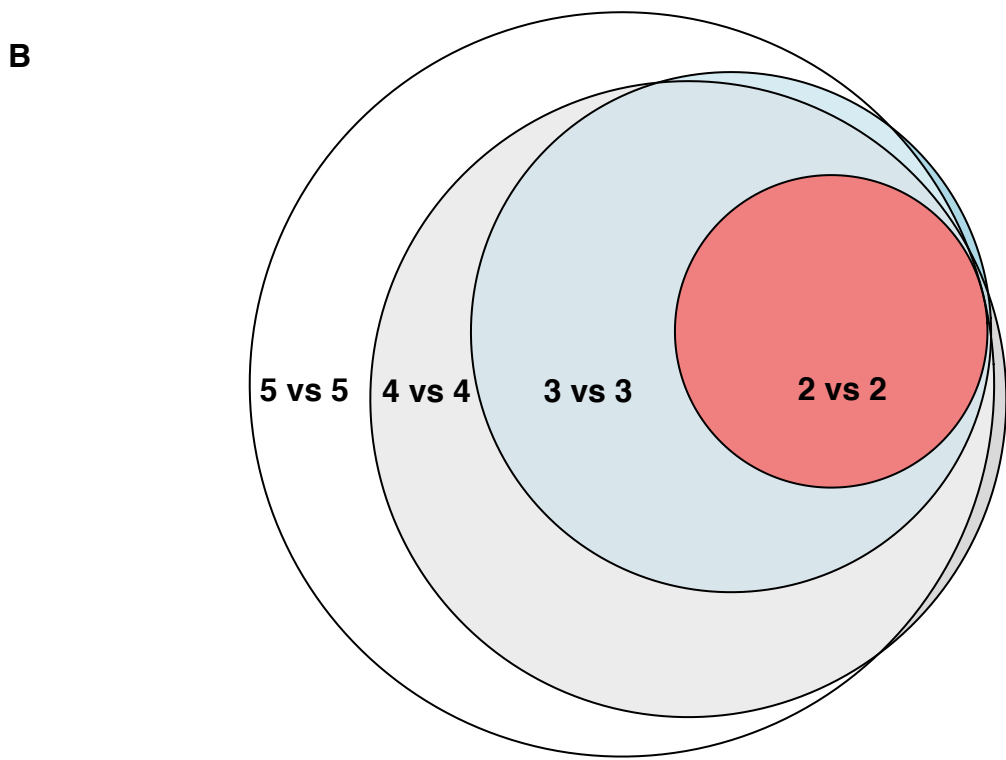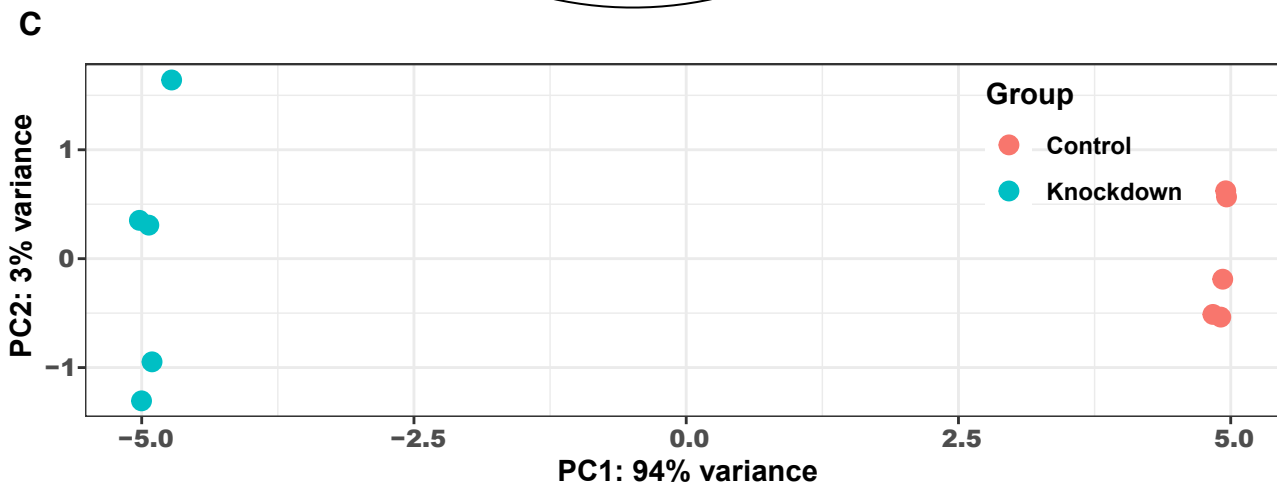

Supplement: S1 Fig — (A) Shown is the number of differentially expressed genes (adjusted p-value<0.01) identified by RNA-seq using 2 to 5 replicates of control and HOXC6 siRNA-treated 22 Rv1 cells. (B) Shown is a four-way Venn diagram displaying the overlap of differentially expressed genes (adjusted p-value <0.01) identified by RNA-seq using 2 to 5 replicates of control and HOXC6 siRNA-treated cells. (PDF) [file pone.0228590.s001.pdf]

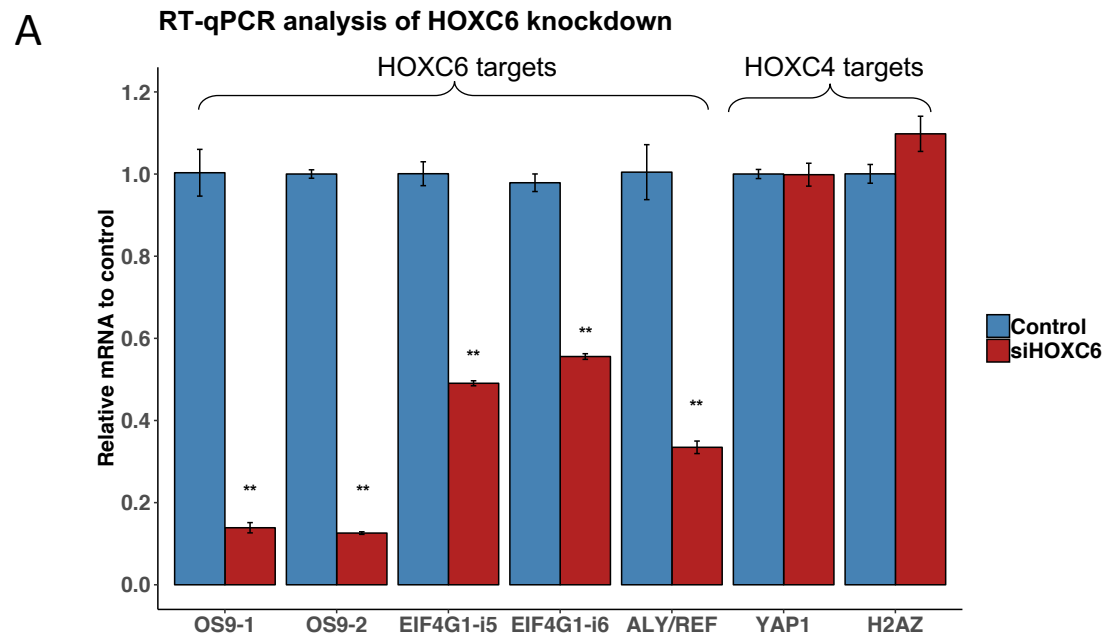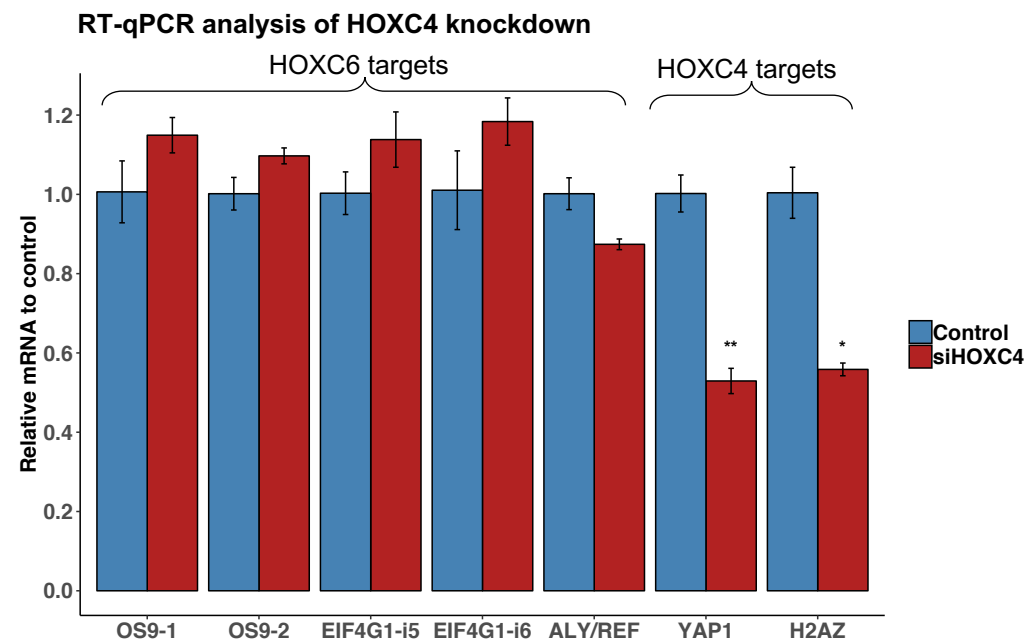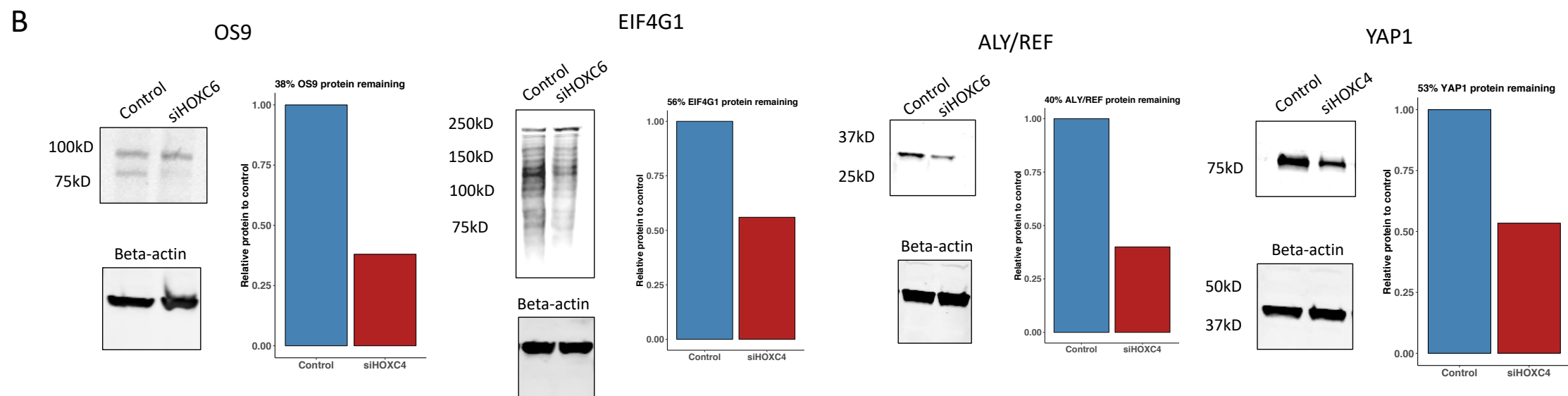

S2\_Fig

Supplement: S2 Fig — (A) RT-PCR analysis of HOXC4- and HOXC6-regulated genes. A second siRNA knockdown of HOXC4 or HOXC6 was performed using 22Rv1 cells. In each panel, the genes identified to be regulated by the specific HOXC protein are indicated; the genes identified as targets for the other HOXC protein are used as controls. (B) Western blot confirmation of HOXC4- and HOXC6-regulated genes; OS9, EIF4G1, and ALY/REF were identified by RNA-seq as HOXC6-regulated genes, whereas YAP1 was identified by RNA-seq as a HOXC4-regulated gene. (PDF) [file pone.0228590.s002.pdf]

**Data generation**

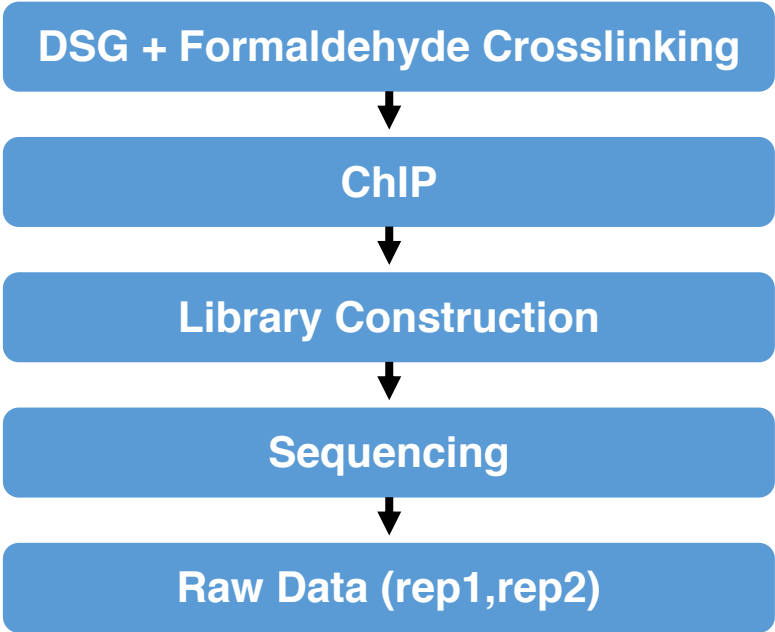

**Peak identification**

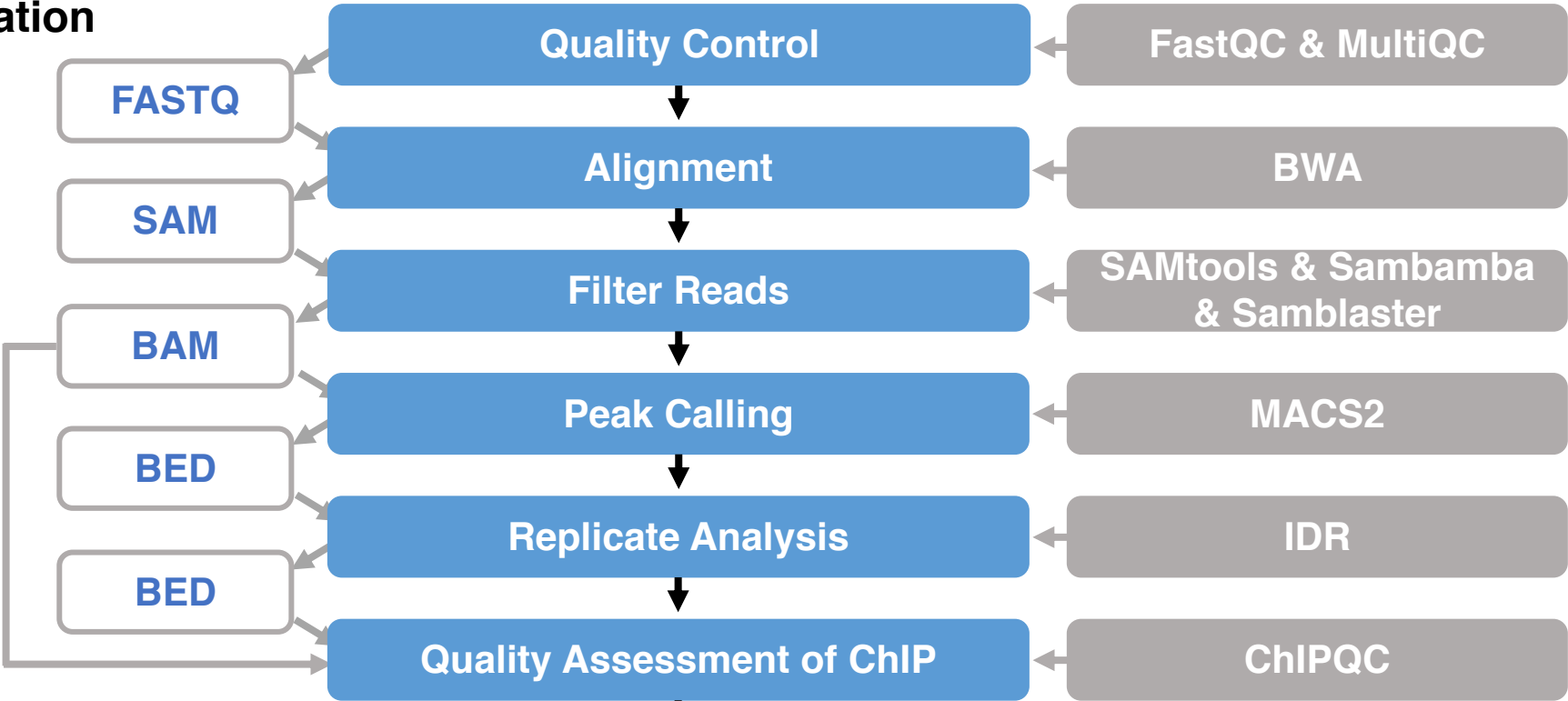

**Biological interpretation**

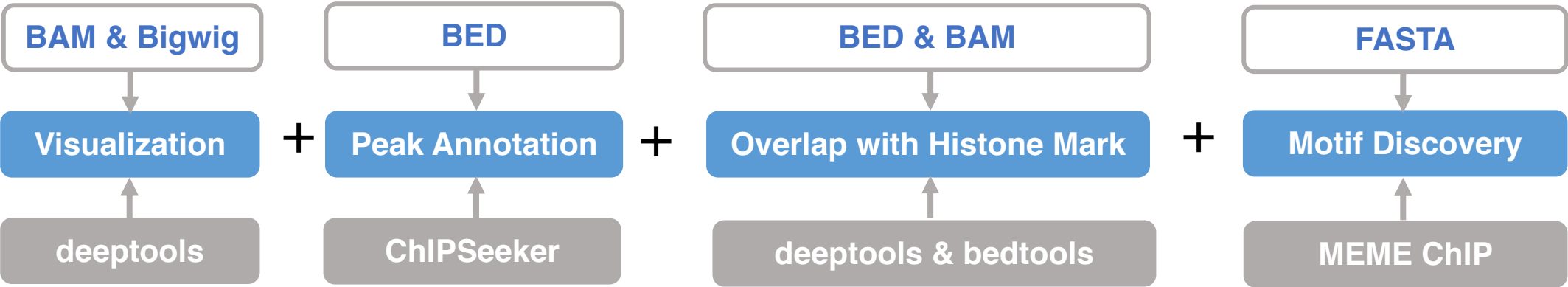

Supplement: S3 Fig — Shown are the steps used to perform and analyze the HOXC6 ChIP-seq experiments; see Methods for details. (PDF) [file pone.0228590.s003.pdf]

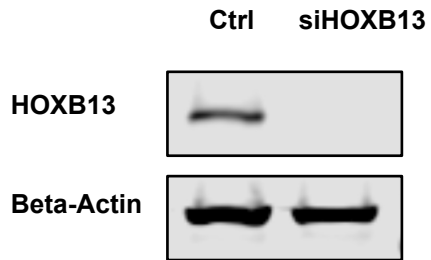

**S4\_Fig**

Supplement: S4 Fig — Shown is a Western blot demonstrating the specificity of the HOXB13 antibody; siRNA-mediated knockdown of HOXB13 mRNA eliminates the signal detected by the HOXB13 antibody. (PDF) [file pone.0228590.s004.pdf]
