# Supplementary material for: Genome-wide analysis of HOXC4 and HOXC6 regulated genes and binding sites in prostate cancer cells
Source: PLoS One. 2020 Feb 3;15(2):e0228590. doi: 10.1371/journal.pone.0228590 (PMC6996832; doi:10.1371/journal.pone.0228590)
Supplement: S5 Fig — Shown are 3 tests that measure the overlap between the binding sites of HOXC6, HOXC4, HOXB13, FOXA1 and AR. The yellow number is the P-value for a two tail fisher exact test obtained using the bedtools fisher function, the red number is the Jaccard value generated using the bedtools jaccard function, the blue value is the number of overlapped peaks called using the MACS2 peak caller. (PDF) [file pone.0228590.s005.pdf]

	HOXC6	HOXC4	HOXB13	FOXA1	AR
HOXC6	0<br>1<br>13729	0<br>0.0739625<br>5654	0<br>0.06782<br>1694	0<br>0.0708701<br>2356	0<br>0.0656927<br>3137
HOXC4		0<br>1<br>12000	0<br>0.0424795<br>1649	0<br>0.0254628<br>2613	0<br>0.0266897<br>2912
HOXB13			0<br>1<br>6457	0<br>0.140703<br>3430	0<br>0.113955<br>3768
FOXA1				0<br>1<br>12951	0<br>0.192298<br>7967
AR					0<br>1<br>30000

P value: Fisher exact test   Jaccard   Number of overlapped peaks

S5_Fig
